# Stochastic and deterministic drivers of seasonal variation of fungal community in tobacco field soil

**DOI:** 10.7717/peerj.6962

**Published:** 2019-06-14

**Authors:** Xing Li, Tianming Li, Delong Meng, Tianbo Liu, Yongjun Liu, Huaqun Yin, Jie Deng, Songrong Zeng, Li Shen

**Affiliations:** 1 School of Minerals Processing and Bioengineering, Central South University, Changsha, China; 2 Key Laboratory of Biometallurgy, Ministry of Education, Changsha, China; 3 College of Agronomy, Hunan Agricultural University, Changsha, China; 4 School of Ecological and Environmental Sciences, East China Normal University, Shanghai, China; 5 Hery Fok Collge of Life Sciences, Shaoguan University, Shaoguan, China

**Keywords:** Seasonal variation, Fungal community, Community assembly, Ecological processes, Stochastic process, Dispersal

## Abstract

**Background:**

The soil fungal community plays an important role in global carbon cycling and shows obvious seasonal variations, however, drivers, particularly stochastic drivers, of the seasonal variation in the fungal community have never been addressed in sufficient detail.

**Methods:**

We investigated the soil fungal community variation between summer growing (SG) and winter fallow (WF) stage, through high throughput sequencing of internal transcribed spacer (ITS) amplicons. Subsequently, we assessed the contribution of different ecological processes to community assembly using null-model-based statistical framework.

**Results:**

The results showed that the fungal community diversity decreased significantly after tobacco cropping in the SG stage and the composition showed a clear turnover between the WF and SG stages. The variation in community composition was largely attributable to the presence of a small portion of Dothideomycetes in the WF stage that dominated the soil fungal community in the SG stage. The organic matter, temperature, and water content were the main deterministic factors that regulated the fungal community; these factors explained 34.02% of the fungal community variation. Together with the result that the fungal community was mainly assembled by the dispersal process, our results suggested that the stochastic factors played important roles in driving the seasonal variation of fungal community. The dispersal limitation dominated the fungal community assembly during the WF stage when homogenizing dispersal was the main assembly process of the fungal community in the SG stage. Thus, we proposed that the dispersal processes are important drivers for seasonal variation of fungal community in tobacco planted soil.

## Introduction

Soil fungal communities are important for qualitative and quantitative of soil organic matter (OM) (Baldrian et al., 2012; Schadt et al., 2003). The fungal communities mediate key processes that control soil carbon cycling and therefore, play an important role for crop production. Substantial studies have indicated that the soil microbial community showed obvious temporal variations (Goss-Souza et al., 2017; Pereira e Silva et al., 2012; Voriskova et al., 2014). They also suggest that revealing the temporal dynamics of fungal community in agriculture system have important significance for understanding the belowground ecosystem, preventing plant diseases, and improving crop productions (Jiang et al., 2018; Leblanc, Kinkel & Kistler, 2015; Xu et al., 2012). Thus, it is of fundamental importance to reveal the seasonal variation of fungal communities in agriculture systems.

It is widely accepted that deterministic factors drive the seasonal variation of fungal community (Gilbert et al., 2012; Voriskova et al., 2014; Zak et al., 2003). Environmental conditions are referred to as the deterministic factors. Different substrates available seasonally can drive seasonal variation of fungal communities (Broeckling et al., 2008; Davey et al., 2012), because dead plant material is available during the winter stage, whereas mainly root exudates are available during the summer stage. In agriculture systems, human activities, including crop planting, and harvesting would strongly affect the chemistry and quality of plant inputs to soil (Tiemann et al., 2015), therefore, the microbial communities in agriculture soils showed obvious differences between seasons (Davey et al., 2012; Pereira e Silva et al., 2012). In addition, climatic conditions such as temperature were also deterministic drives of seasonal variation of fungal community.

Despite the increasing evidences that microbial communities showed seasonal variations, as do the deterministic factors drive, stochastic drivers of the seasonal variation in fungal communities has been rarely studied (Cline & Zak, 2014). Understanding the processes that rule microbial community assembly is a central issue in ecology research. Traditionally, the microbial community is thought to be assembled by “niche-based” (deterministic) mechanisms. Species traits, interspecies interactions, and environmental conditions are often referred to as deterministic processes. However, a large amount of variation in microbial community structure could not be well explained by extensive measurements of all routinely measured environmental variables (Zhou et al., 2008). Recently, the “neutral (stochastic) theories” have gained much more attention (Stegen et al., 2012, 2013a, 2013b; Wang et al., 2013; Zhou & Ning, 2017). It is assumed that the communities are also governed by stochastic processes including birth, death, colonization, extinction, and speciation. A null-modeling-based statistical framework has been built up by Stegen et al. (2012, 2013b) to identify the importance of deterministic and stochastic factors on the assembly of soil microbial communities. Because the null model approaches have various options of algorithms, the approaches are flexible compared to neutral models. In addition, it could be easier to develop a statistical framework based on null model approaches to disentangle the influences of different processes, as the null model approaches can be based on taxonomic and phylogenetic diversity, or be extended to functional diversity metrics. Even though the framework does not parse out sub-classes of selection, such as competition and trophic interactions, it estimates the relative influences of ecological processes. In the null-model-based framework, assembly of microbial community is governed by four processes that are selection, dispersal, drift, and mutation (Zhou & Ning, 2017). The selection includes the effects of abiotic conditions (environmental factors) and biotic interactions (e.g., competition and trophic interactions, etc.), and is the deterministic process. The drift is the random changes in organism abundance caused by the inherent stochastic processes of birth, death, and reproduction. The dispersal and mutation may include both deterministic and stochastic factors. The dispersal is the movement and successful colonization of organisms from one location to another via active or passive mechanisms. It is suggested that microbial community is assembled through the combination of both niche and neutral processes (Dumbrell et al., 2010; Zhou & Ning, 2017). Although our understanding of the assembly of microbial community is broadly expanded, debates on the main ecological process governing microbial community continue. Goss-Souza et al. (2017) found that the grassland soil microbial community assembly was mainly ruled by stochastic processes, whereas, Dumbrell et al. (2010) suggested that although the soil arbuscular mycorrhizal fungal community was influenced by stochastic processes, the communities were assembled in a predictable manner. However, the deterministic and stochastic drivers of seasonal turnover in fungal community in agriculture system have never been addressed in sufficient detail.

In the present study, we analyzed the soil fungal communities at the winter fallow (WF) and summer growing (SG) stages, through Illumina Miseq sequencing of internal transcribed spacer (ITS) region. In addition, we used a null-modeling-based statistical framework to quantify the contributions of various ecological processes to the fungal community. We aimed to reveal the seasonal variation of the fungal communities in agriculture systems. We also hypothesized that the stochastic process played a role in the seasonal variation of soil fungal communities in agricultural systems. The results in this study indicated that differences in the fungal community were mainly caused by the differences in the community assembly processes.

## Materials and Methods

### Site description and soil sampling

The experiment site is located in Xiangxi (109°27′E and 28°30′N), China. The area has an annual mean temperature of 16–18.5 °C and has an annual rainfall of 1,500 mm. The tobacco was planted during March and July. Fertilizations were applied when tobacco were planted and fertilization amount was 1,350 kg ha^−1^ with the N:P:K (N, P_2_O_5_, and K_2_O, respectively) ratio of 1:1.2:2.43. After the tobacco was harvested, the field was fallow until next March. Soil samples were collected in two stages: the WF stage and the SG stage, respectively. WF samples were collected before tobacco transplanting (March 27th, 2014) and SG samples were collected before tobacco harvesting (July 28th, 2014). To obtain replicated samples, the site was divided into ten equivalent plots (10 × 10 m^2^ each). The top layer soils (0–20 cm) were collected using the checkerboard method. In the checkerboard method, each field was divided into five areas (each 10 × 2 m^2^) and five soil cores were taken from each area and homogenized by mixing. The samples from each area were then separated into two parts: one portion was frozen in liquid nitrogen immediately and stored in –80 °C before DNA extraction. The other portion was taken into laboratory for soil physical and chemical determinations.

Soil temperature was measured in situ using a soil thermometer. Soil pH was measured by homogenizing five g air dried soils in 25 ml sterile distilled water and determined using a pH meter (Portable ORP meter, BPH-220, Bell, China). Soil OM and total nitrogen (TN) were analyzed as described in our previous study (Li et al., 2017). The Potassium (K), Iron (Fe), and Calcium (Ca) were analyzed by Inductively Coupled Plasma—Atomic Emission Spectroscopy.

### DNA extraction, PCR amplification, and sequencing

Soil genomic DNA was extracted using the MO BIO PowerSoil DNA Isolation Kit (MO BIO, SanDiego, CA, USA) following the manufacturers’ instructions. The fungal ITS were amplified using primer pairs ITS7F (5′-GTGARTCATCGARTCTTTG-3′) and ITS4R (5′-TCCTCCGCTTATTGATATGC-3′). PCR was performed on a Biosystems 2720 Thermal Cycler (ABI Inc., Shanghai, China) with the ITS primer pair with barcodes and specific sequencing adapters. The 50 μl PCR reagents included 25 μl of TaqMaster Mix (Vazyme, Piscataway, NJ, USA), 2.0 μl DNA (∼20 ng/μl), one μl primers (10 nM each) and 22 μl DNase-free deionized water. PCR was set as follows: 94 °C denaturing for 5 min, followed by 35 cycles of 94 °C for 30 s, 58 °C annealing for 45 s, and 72 °C for 30 s, and a final step of extension at 72 °C for 10 min. PCR products were recovered using the E.Z.N.A. TM Gel Extraction Kit (OMEGA Biotek Inc., Doraville, GA, USA) following the manufacturers’ instructions. Equal amounts of PCR product from all samples were mixed together and was quantified using the Qubit 2.0 Fluorometer (Life Technologies, Carlsbad, CA, USA). The 2 × 250 bp paired-ends sequencing was performed on Miseq sequencing platform (Illumina, San Diego, CA, USA) using the Miseq 500 cycles kit. The raw data is FASTQ format. The Illumina-specific sequences were cut off before data processing.

### Processing sequencing data

The raw data was processed on the Galaxy pipeline developed by the Institute of Environmental Genomics, University of Oklahoma (http://zhoulab5.rccc.ou.edu:8080/root), as described in our previous studies (Li et al., 2017; Meng et al., 2019). Firstly, reads were assigned to different samples according to their barcode sequences and then the barcode and primer sequences were removed. Subsequently, forward and reverse reads were merged into full-length amplicon sequences with 30–250 bp overlapping, using the Flash (Version 1.0) (Magoc & Salzberg, 2011). For quality filtering, low quality sequences (QC score < 20 and length < 160 bp) were trimmed using Btrim (Version 1.0) (Kong, 2014), sequences containing “N” were also trimmed. The chimeras were checked and removed by aligning sequences to ITS warcup reference sequences. Finally, sequences with 97% identity were assigned to the same operational taxonomic unit (OTU) using UPARSE (version usearch v7.01001_i86linux64) (Edgar, 2013), and singletons that have no similar sequences were removed. We obtained 11,586–153,463 clean sequences per sample and rarefied all samples by randomly choosing 11, 000 sequences for each sample. The raw data was deposited to the NCBI, bioproject accession number PRJNA515768 (https://www.ncbi.nlm.nih.gov/sra/?term=PRJNA515768).

### Microbial community analysis

Shannon, Simpson and Pielou evenness indexes were calculated on R statistical platform (version 3.4.0) with package “vegan” (version 2.4-3). The observed OTU number was calculated by counting the observed OTUs in each sample. The Chao1 diversity index was calculated according to the following equation:

(1) $${\rm{chao}}1 = {\rm{Sobs}} + {{F{1^2}} \over {2F2}}$$

Where Sobs is the observed OTU number, *F*1 is the number of singletons and *F*2 is the number of doubletons.

Non-metric multi-dimensional scaling (NMDS) and analysis of similarity (ANOSIM) were performed using “vegan” R package. Fungal community dissimilarity in NMDS was calculated based on the Bray–Curtis distance matrix. Environmental factors’ correlation was tested using “envfit” function in the “vegan” package. Significance tests were performed through student *t*-test, and *p*-values were adjusted using “Bonferroni” method. OTU taxonomic assignment was performed by blasting representative sequences of each OTU to the fungal ITS warcup database in the Ribosomal Database Project (Cole et al., 2009; Wang et al., 2007). When the blasting similarity was less than 50%, the OTU was assigned to “Unclassified”. Linear discriminant analysis (LDA) effect size (LEfSe) (Segata et al., 2011) was used to detect significant different taxa (biomarkers) in different stages. Taxa that have the LDA score > 3 were considered the biomarkers.

### Ecological process analysis

A phylogenetic tree of all OTUs was constructed using Usearch for any further phylogenetic analysis. We used the mean nearest taxon distance (MNTD) (Chu et al., 2016; Stegen et al., 2012) to quantify the phylogenetic turnover in community composition between a pair of samples (i.e., “phylobetadiversity”). The nearest taxon index (NTI, (Webb et al., 2002)) which is the negative output of standardized effect size measure (ses.MNTD), is used to test for phylogenetic clustering or overdispersion. A mean NTI taken across all communities that is significantly different from zero indicates clustering (NTI > 0) or overdispersion (NTI < 0) on average. The MNTD and NTI were calculated using the “picante” R packages.

Moreover, the contributions of various ecological processes to fungal community assembly were inferred by null-model-based statistical framework. To test which community assembly process can best explain the microbial community assembly, we calculated the null-model-based phylogenetic and taxonomic βMNTD and βNTI (Stegen et al., 2013b). The βMNTD represents the dissimilarity between communities and the negative values of differences between βMNTD and the mean of null distribution is referred to as βNTI. The βNTI together with the Bray-Curtis-based Raup-Crick (RC) was used to quantify the contribution of major ecological processes to the assembly of fungal communities. When the |βNTI| > 2, the community assembly is driven by deterministic processes, such as homogeneous selection (βNTI < −2) or heterogeneous selection (βNTI > 2). When the |βNTI| < 2, the community is mainly assembled by stochastic processes, such as dispersal (|RC| > 0.95, including homogenizing dispersal (RC < −0.95) and dispersal limitation (RC > +0.95)) or undominated processes (|RC| > 0.95, including diversification, drift or others).

## Results

### Soil properties

Soil properties including pH, temperature (T), potassium (K), Calcium (Ca), Magnesium (Mn), Iron (Fe), Water content, OM, and TN are shown in Table 1. Soil pH, K, Ca, and Mn did not show obvious differences between the two stages. Soil OM, TN, and Fe were significantly higher at the WF stage than at the SG stage, whereas soil T and water content were significantly higher at the SG stage than the WF stage.

**Table 1:** Soil properties during summer growing (SG) and winter fallow (WF) stages.

	SG	WF
pH	5.10 ± 0.61	5.10 ± 0.46
T, °C	27.24 ± 0.41	13.46 ± 0.14*
K, g/Kg	13.48 ± 2.69	11.94 ± 1.27
Ca, g/Kg	1.24 ± 0.49	1.54 ± 1.08
Mn, mg/Kg	1,250.2 ± 310.7	1,310.4 ±7 8.5
Fe, g/Kg	24.98 ± 6.73	34.62 ± 8.68*
Water content, %	15.96 ± 1.04	0.25 ± 0.03*
OM, g/Kg	14.83 ± 0.91	18.13 ± 1.09*
TN, g/Kg	2.43 ± 0.12	2.71 ± 0.16*

**Notes:**

Results are means and S.D of 10 replicates. T, Temperature; OM, Organic matter; TN, total nitrogen.

* indicates the difference between stages is significant at *p* < 0.05 level.

### Soil microbial community

**Figure 1:**
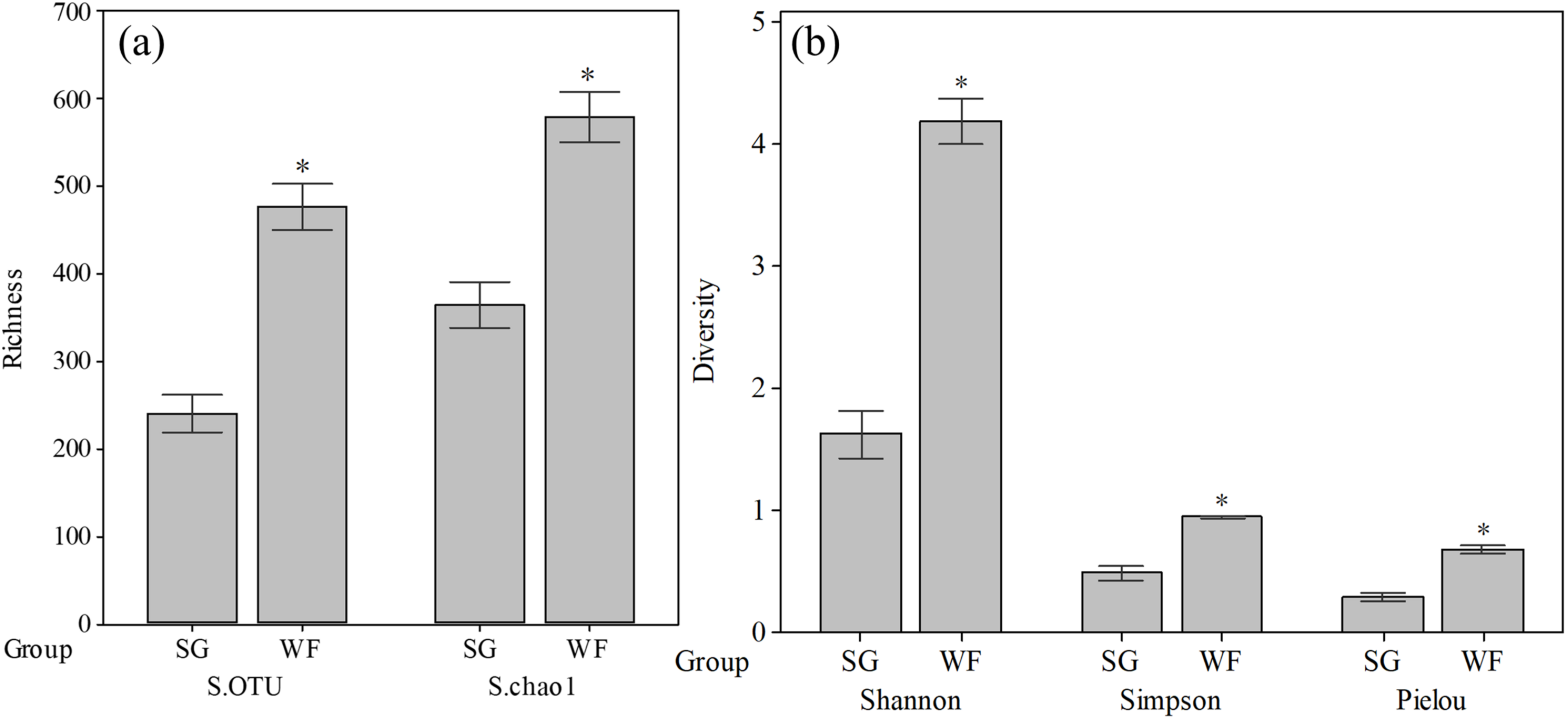
Fungal community richness and diversity at SG and WF stages. (A) Richness. (B) Diversity indices. Diversity indices were calculated using “vegan” R package. The asterisks above the WF columns indicate the differences between the SG and WF stages is significant at *p* < 0.05 as indicated by Student *t*-test. S.OTU: observed OTU number.

We obtained 542,736 high quality sequences for all 20 samples, which could be classified to 2,165 OTUs. To remove any biases caused by different sequencing depths, we resampled all samples by randomly choosing 14,479 sequences. The rarefaction curves showed that increasing the sequencing depth would not cause an obvious increase in observed OTUs after rarefying, indicating the sequencing depth is sufficient for downstream analyses. We observed more OTUs at the WF stage soils than at the SG stage soils (Fig. 1A). The Chao1 index also suggested that the fungal community at the WF stage possessed higher richness. The soil fungal community diversity indexes including Shannon, Simpson, and Pielou diversity, were significantly higher at the WF stage than that at SG stage (Fig. 1B).

**Figure 2:**
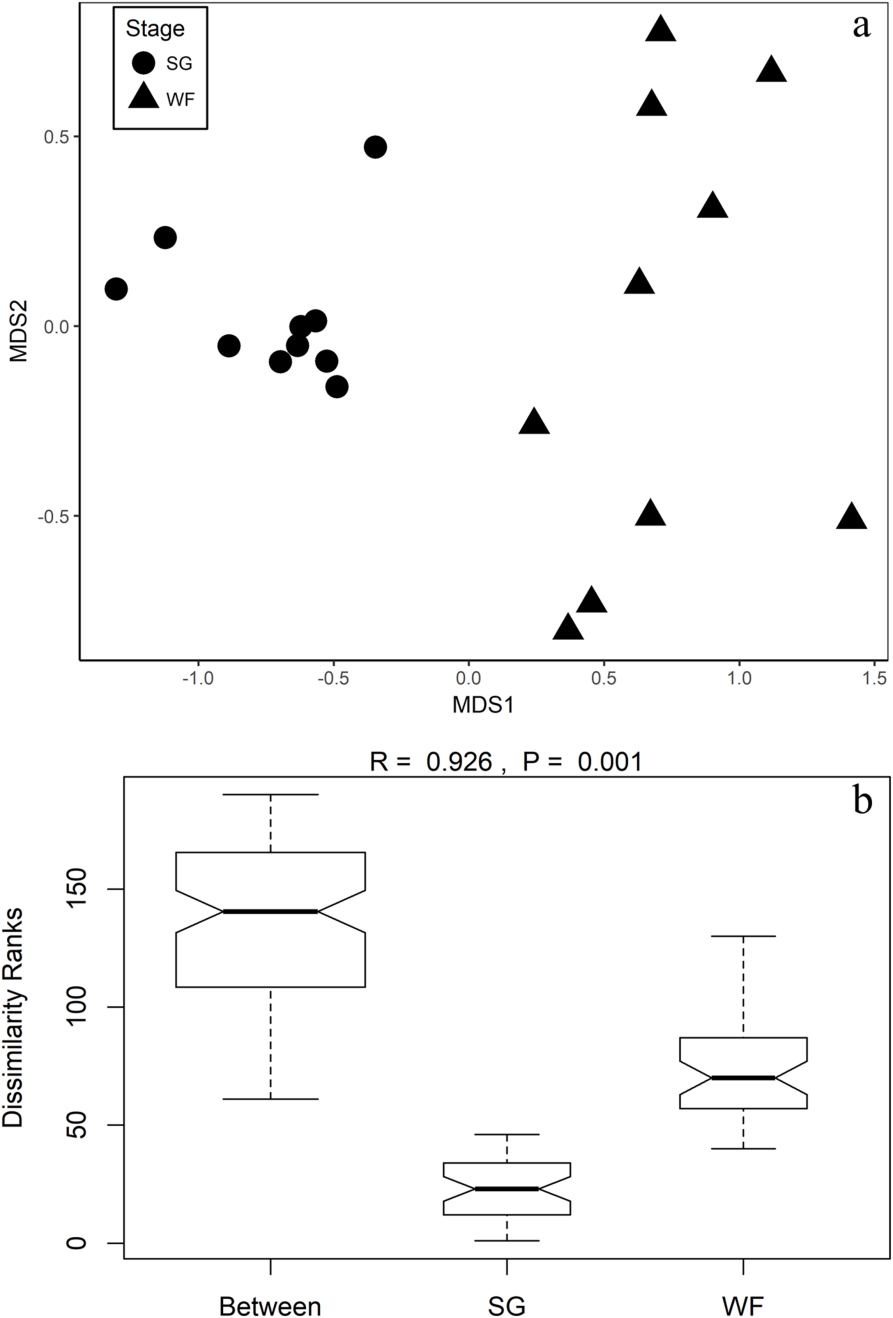
NMDs and ANOSIM shows the fungal community composition differences between the SG and WF stage. (A) NMDS. (B) ANOSIM. NMDS and ANOSIM were calculated based on the Bray–Curtis distance matrix using “vegan” R package.

To examine the fungal community composition differences between the SG and WF stages, we carried out the NMDS analyses and ANOSIM. The NMDs plot showed that SG samples and WF samples were clearly separated from each other, indicating the fungal community composition differed obviously between the SG and WF stages (Fig. 2A). In addition, the SG samples were more closely grouped than the WF samples. ANOSIM (Fig. 2B) further confirmed that the differences in fungal community composition between the SG and WF stages were significant (*p* = 0.001).

### Phylogenetic analysis of microbial community

**Figure 3:**
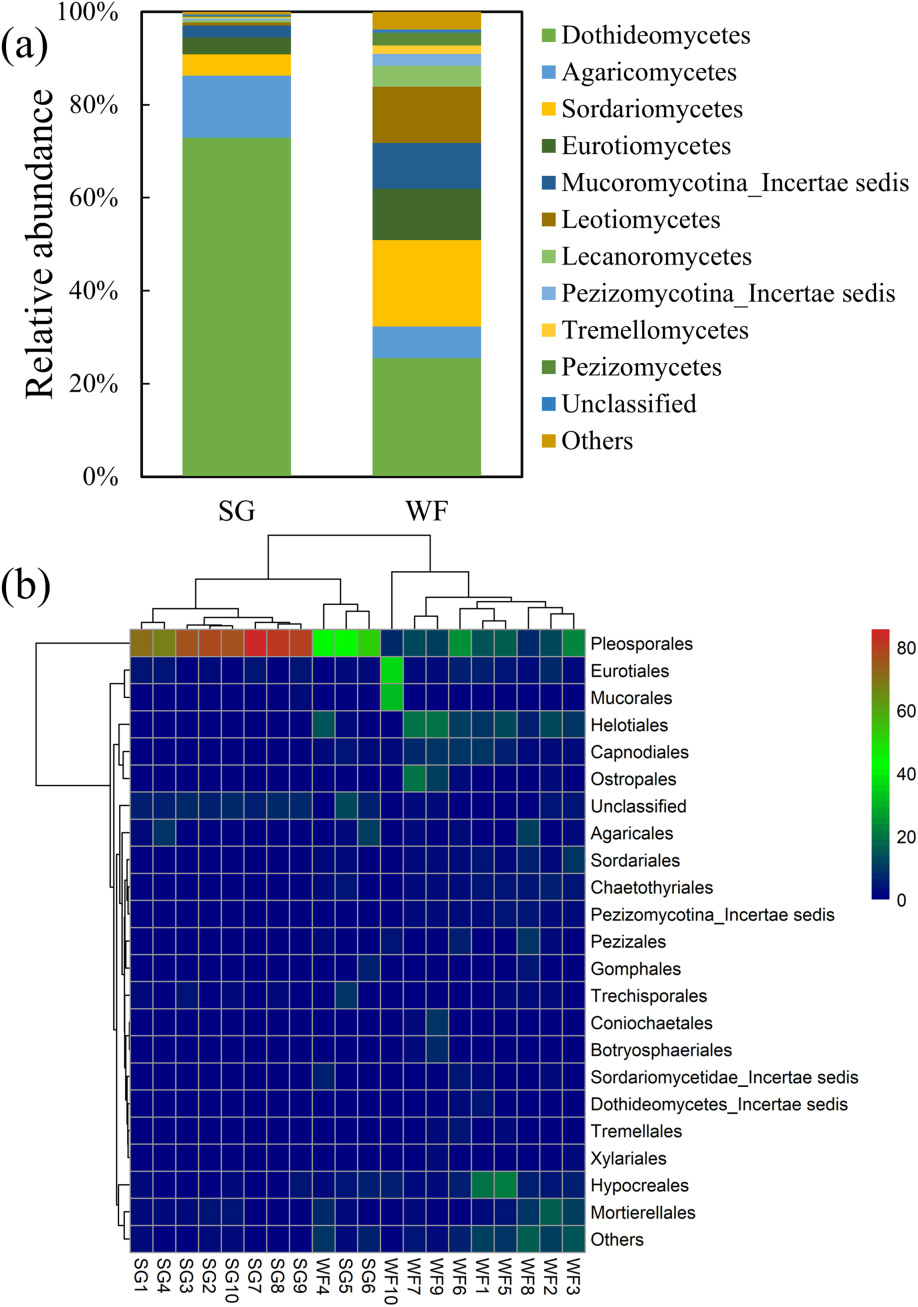
Fungal community composition at Class and Order level. (A) Bar charts at Class level. (B) Heatmap at Order level. Others included taxa with relative abundance of <0.5%.

**Figure 4:**
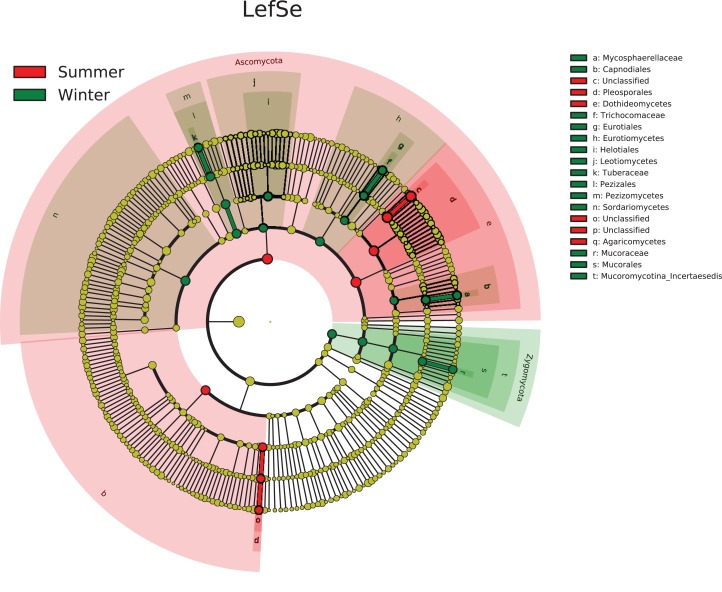
Linear discriminant analysis (LDA) effect size (LEfSe) analysis showing fungal taxa distribution differences between the summer growing (SG) and winter fallow (WF) stages. The taxa that has the LDA score > 3.0 is considered the biomarker.

We detected five phyla with 196 genera in all samples. At the phylum level, the fungal community was dominated by Ascomycota that did not differ obviously between stages. The distribution pattern of fungal community showed obvious differences between SG stage and WF stage at class level. During the SG stage, the soil fungal community was dominated by Dothideomycetes of phylum Ascomycota that account for more than 70% of the fungal community, whereas during the WF stage, the fungal community distributed more evenly (Fig. 3A). The heatmap (Fig. 3B) showed that the SG and WF (except for WF4) samples clustered separately, which suggested they differed obviously in community composition at the order level. Similar to the composition at the Class level, the SG samples were dominated by one order, the Pleosporales, whereas the fungal community distributed evenly at the WF stage. To understand which fungal taxa differed between different stages, we carried out LDA effect size (LEfSe) analysis (Fig. 4). The taxa that has the LDA score > 3.0 is considered the biomarker. LEfSe indicated that the taxa showed significant difference between the SG and WF stages. The Dothideomycetes (class), Agaricomycetes (class), and Pleosporales (order) were significantly enriched in the SG stage, whereas, Zygomycota (phylum), Mucoromycotina_Incertaesedis (class), Mucorales (order), Mortierellales (order), Mucoraceae (family), Mortierellaceae (family), Tremellomycetes (class), Tremellales (order), and Tremellaceae (family) were significantly enriched in the WF stage. In addition, there some taxa in phylum Ascomycota showed obvious enrichment in the WF stage.

### Deterministic and stochastic processes

**Figure 5:**
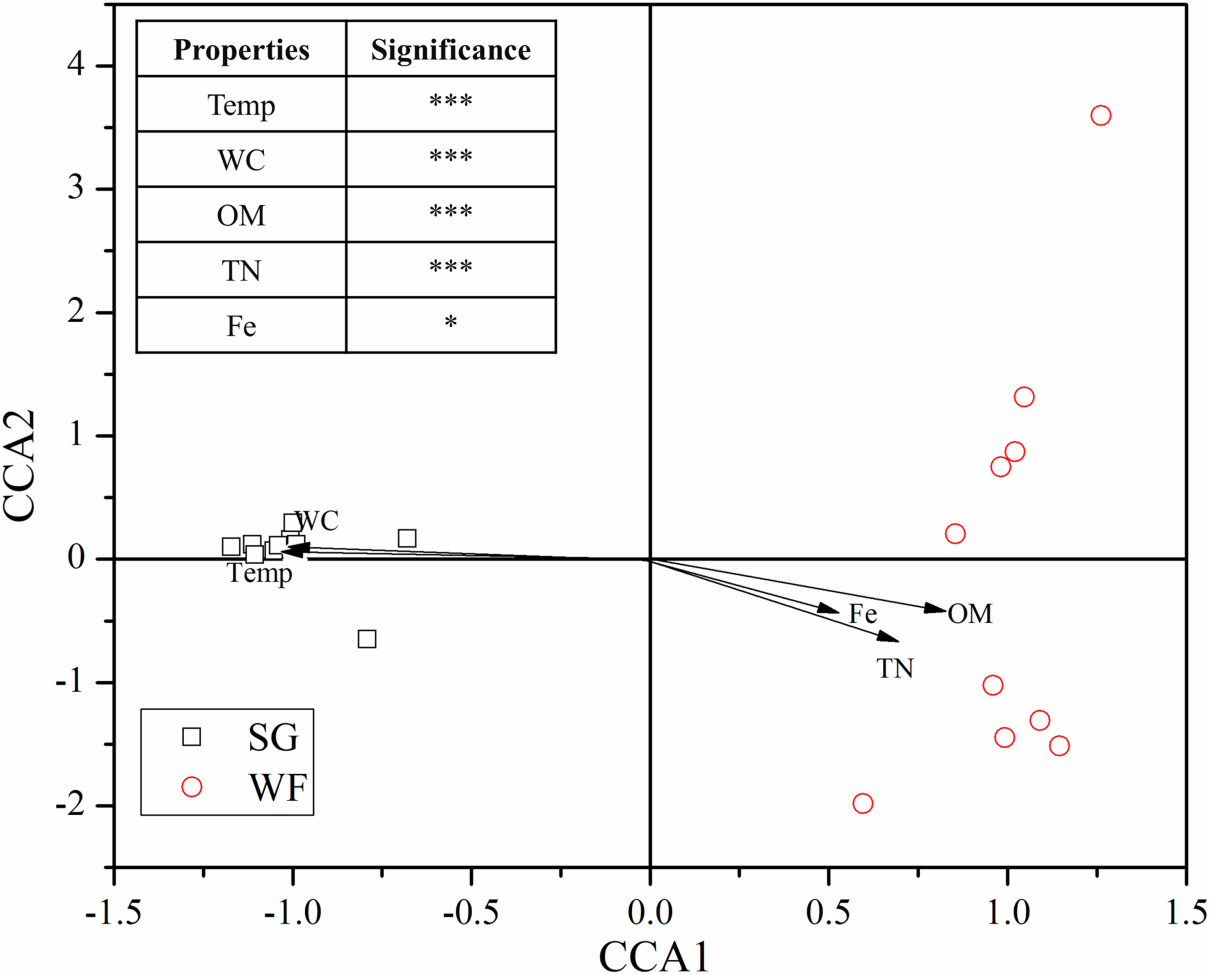
Canonical correspondence analysis (CCA) shows main factors regulating soil fungal community. CCA was performed using “vegan” R package. Temp, Temperature; WC, Water content; OM, Organic matter; TN, Total nitrogen; *p < 0.05, ***p < 0.001.

In order to examine influence of the local environmental factors on microbial community, we carried out Pearson correlation and CCA analysis which indicated the investigated soil properties constrained the fungal community significantly (*F* = 1.44, *p* = 0.02). Among the parameters, soil temperature, water content, OM, TN, and iron content showed significant correlation with soil fungal community diversity (Table S1), which could well explain the fungal community dissimilarities (Fig. 5). Taken together, the variables explained 34.02% of the fungal community variation.

**Figure 6:**
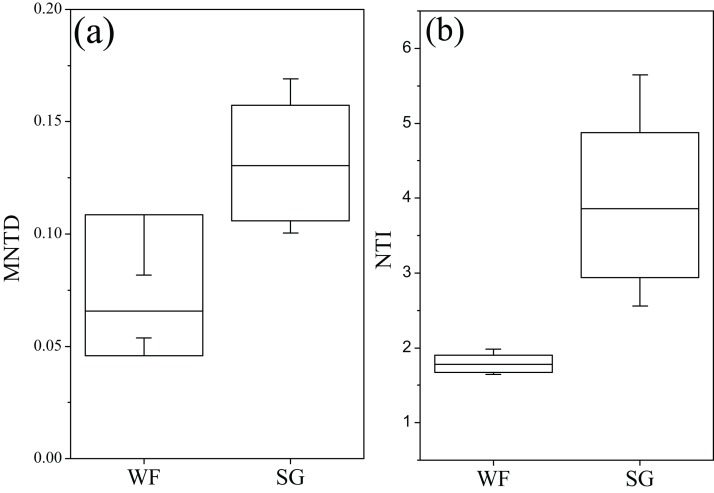
Variation of mean nearest taxon distance (MNTD) and the nearest taxon index (NTI) of fungal community during summer growing and winter fallow stage. (A) MNTD. (B) NTI. MNTD and NTI were calculated using “picante” R package.

In order to analyze the phylogenetic community composition, we calculated MNTD and NTI (Fig. 6). The MNTD values were significantly (*t*-test, *p* < 0.001) higher in the WF stage than in the SG stage, which suggested the larger variations of phylogenetic community composition in the WF stage, compared to the SG stage. Mean NTI values of both WF and SG stages were significantly larger than 0, indicating the fungal communities tended to be phylogenetically clustered, rather than overdispersion on average. In addition, all individual NTI values of the WF stage were greater than +2, indicating coexisting taxa at the WF stage were more closely related than expected by chance (phylogenetic clustering).

**Figure 7:**
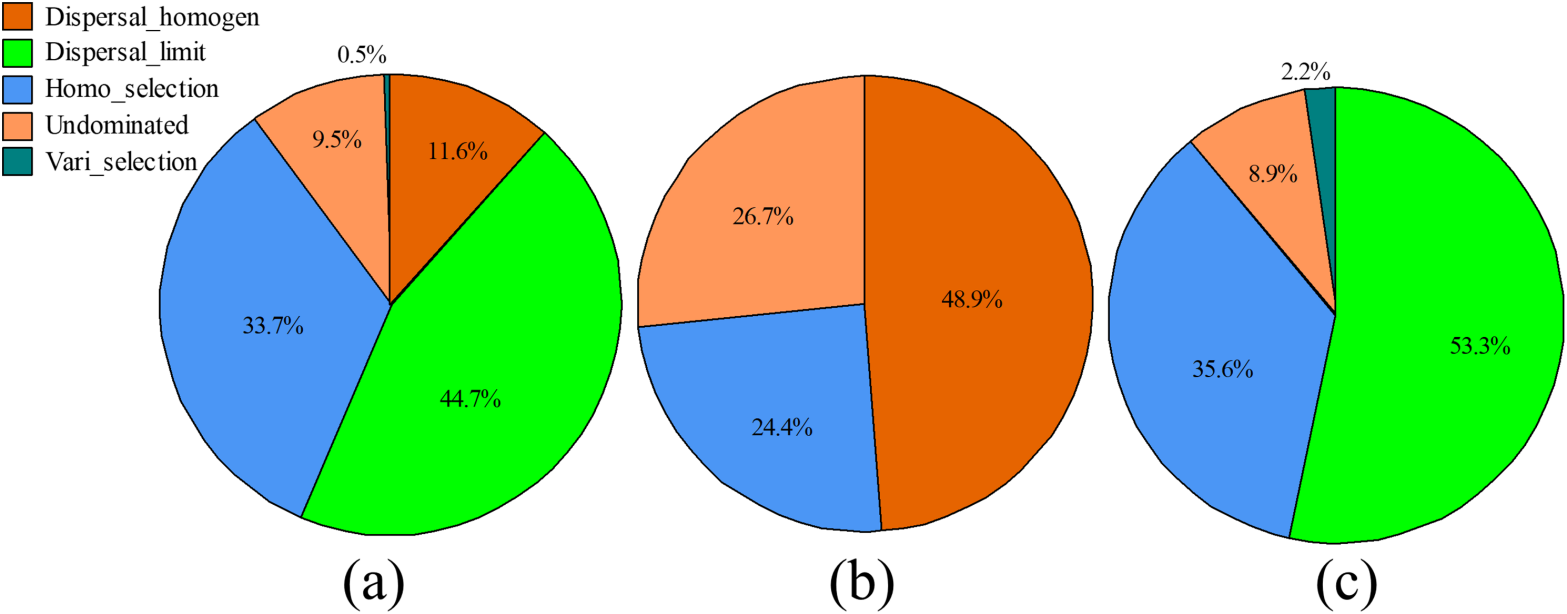
Contribution of ecological processes that determine fungal community assembly. (A) In general. (B) Summer growing stage. (C) Winter fallow stage.

Furthermore, most of the βNTI has an absolute value of <+2 indicating a large proportion of stochastic events in the fungal community. Generally, selection contributed to 34.2% of the community assembly (homogeneous selection contributed 33.7% and heterogeneous selection contributed to 0.5%), whereas, assembly of the fungal community was dominated by dispersal process with dispersal limitation contributed 44.7% and homogenizing dispersal contributed 11.6% (Fig. 7). Even though the dispersal plays an important role in the assembly of fungal community in soils in both the SG and WF stages, homogenizing dispersal dominates the subassembly of fungal community in the SG stage, while dispersal limitation is much more pronounced in the WF stage.

## Discussion

Soil conditions, including soil temperature, pH, and OM contents are generally accepted as the main factors governing soil microbial community (Li et al., 2017). During the last decades, the relationship between aboveground plant diversity and belowground fungal community was also widely studied (Waldrop et al., 2006). Our results further supported the role of these factors in regulating soil fungal communities. As indicated by the CCA and correlation analysis, the soil temperature, OM contents, pH, and Ca and Fe contents play a significant role in shaping the soil fungal community, which is in agreement with the previous studies (Hu et al., 2018; Sheng et al., 2013). Taken together, 34.02% of the variations in fungal community could be explained by an investigation of the factors. In the community assembly framework, we also found that the community was influenced by the deterministic processes, that is, selection. The selection, including both homogeneous and heterogeneous selection, ruled 34.2% of the fungal community assembly. We also observed that the temperature, water content, OM, soil TN, and Ca and Fe contents were strongly correlated with the fungal community diversity, which further demonstrated the role of deterministic process. In addition, soil fungal communities tended to be phylogenetically clustered, rather than by chance, which is consistent with several previous studies in different habitats (Chu et al., 2016; Stegen et al., 2012; Wang et al., 2013; Yan et al., 2017). The phylogenetic clustering was stronger at the winter stage than at the summer stage, as indicated by the significant greater absolute magnitude of NTI at the WF stage than at the SG stage (larger NTI values represent greater phylogenetic clustering effects). Taken together, the results suggested that the soil fungal communities were partially structured by environmental filtering and the fungal community responded to the abiotic factors in a partially predictable manner.

Recent studies suggested that not only the deterministic abiotic factors, but also the community assembly stochastic processes, acted strongly on the microbial communities (Caruso et al., 2011; Hu et al., 2015; Kinnunen et al., 2018; Zhou & Ning, 2017). In our study, large proportion of fungal community variation could not be well explained by deterministic factors, indicating there were either factors that were not investigated or stochastic drivers that need to be considered. We found that the fungal community was mainly assembled by the dispersal process, including the homogenization dispersal and dispersal limitation. Dispersal is the movement and successfully colonization of organisms across different localizations (Hanson et al., 2012; Vellend, 2010). Theoretically, dispersal can be either deterministic, stochastic, or both, however, it was considered as the stochastic prosses in many studies (Finlay, 2002). Therefore, our results are in agreement with previous studies which suggested that soil microbial community was influenced by the stochastic-neutral processes (Fierer et al., 2012; Stegen et al., 2015).

We also found that the ecological processes were responsible for the variation of fungal communities. The fungal communities are more prone to be influenced by homogenizing disposal after cropping (summer stage). Differently, the winter stage (before cropping) communities have a disposal limitation assembly. The homogenizing dispersal (high dispersal rates) can homogenize the community structure (Zhou & Ning, 2017) and therefore, lead to little variation in the fungal community composition during the summer stage. Conversely, lower dispersal rates (dispersal limitation) suggest microbial populations have a high tendency to migrate and can increase the community variation. Therefore, the community assembly processes are responsible for compositional differences of the fungal community. Serious disturbance would cause significant changes in ecological patterns (Fierer et al., 2012). Human activities in agriculture systems, such as plant cropping and agriculture management, are obvious disturbances to ecosystems. Thus, the human activities might be responsible for the change in fungal community assembly from the WF stage (when no, if any few human activities were applied to the ecosystem) to the SG stage (when crops were planted and fertilizers were applied). Therefore, we propose that human activities have significant effects on fungal community assembly, and therefore, the soil fungal community is affected.

In agriculture systems, the chemistry and quality of plant inputs to soil change across time (Davey et al., 2012), and therefore the belowground microbial communities would change across time. The results demonstrated that the fungal community was very variable between the SG and WF stages, exhibiting obvious changes in diversity and composition. Firstly, the fungal community was significantly more diverse in winter than in summer, which is consistent with several previous studies which found the fungal community had the highest diversity during later winter in forest soils (Schadt et al., 2003; Voriskova et al., 2014). This is because the different substrates available seasonally (Lipson, Schadt & Schmidt, 2002): dead plant material is available during the WF stage, whereas only root exudates are available during the SG stage. This can be also applied to agriculture soils. That is, only root exudates of crop plants were available and weeds were artificially removed during the SG stage, whereas, during WF stage, dead material, and exudates from diverse weeds were available for microorganisms. In addition, compared to the forest, agriculture system has very low plant diversity (i.e., only tobacco was cropped) during the SG stage. Besides, more human activities will be applied to agriculture systems, particularly during the crop growing stage (i.e., SG stage). The human activities, including chemical fertilizations and agriculture management activities during the cropping stage, would have obvious effects on fungal communities. It could also be responsible for the low fungal community diversity during the SG stage. Chemical fertilizers could reduce soil microbial community diversity (Ge et al., 2008; Sun et al., 2016; Zhen et al., 2014), and low plant diversity during monoculture would cause low soil fungal community diversity (Schadt et al., 2003; Zak et al., 2003). Secondly, the NMDS and ANOSIM revealed a complete turnover between the WF stage and SG stage fungal community composition. The summer and winter fungal community showed very different distribution patterns. This is similar to the findings that the summer and winter fungal community had very different compositions (Schadt et al., 2003; Voriskova et al., 2014). The differences in community composition was largely attributable to the presence of a large proportion of Dothideomycetes in the SG stage that had relatively low abundance in the WF stage. Members of the Dothideomycetes are found as endophytes or epiphytes of living plants (Schoch et al., 2006), therefore, the enrichment of Dothideomycetes during the SG stage is the result of crop cultivation.

## Conclusions

We have demonstrated that fungal community in the agriculture system is very dynamic, showing significant seasonal changes in both community diversity and composition. The results showed that fungal community diversity decreased significantly after tobacco cropping in summer stage. The composition of fungal community showed a clearly turnover between the WF and cropping stages, and the WF fungal community is more homogenized than the SG fungal community. The shift in community composition was largely attributable to the presence of a small portion of Dothideomycetes in the WF stage which dominates the fungal community in the summer cropping stage. In addition, the WF community had larger variations in composition than the SG community. The results also showed that 34.02% of the fungal community variation could be well explained by the environmental factors, particularly the soil OM. This proportion was considerable as the deterministic processes contributed to the fungal community variation. The results suggested the stochastic processes (i.e., dispersal) played important roles in the assembly of fungal community in soils. The dispersal limitation dominated the fungal community assembly during the WF stage when homogenizing dispersal was the main assembly process of the fungal community in the SG stage. Therefore, the large variation in the fungal community during the winter stage was a result of dispersal limitation which dominates the fungal community assembly in soils during the WF stage. The results indicated that differences in the fungal community were mainly caused by the differences in the community assembly processes. We also propose that human activities, such as crop cultivation and harvesting have significant effects on fungal community assembly, and therefore, the soil fungal community is affected.

## Supplemental Information

10.7717/peerj.6962/supp-1 Supplemental Information 1 Individual measurements.

10.7717/peerj.6962/supp-2 Supplemental Information 2 Pearson correlation between soil properties and fungal community diversity indices. WC: water content, OM: organic matter, TN: total nitrogen, Temp: Temperature, S.OTU: observed OTU number.

## Additional Information and Declarations

### Data Availability

The following information was supplied regarding data availability: The raw data are available in the SRA database under BioProject accession number is PRJNA515768: https://www.ncbi.nlm.nih.gov/sra/?term=PRJNA515768.

## References

[ref-1] Baldrian P, Kolarik M, Stursova M, Kopecky J, Valaskova V, Vetrovsky T, Zifcakova L, Snajdr J, Ridl J, Vlcek C, Voriskova J (2012). Active and total microbial communities in forest soil are largely different and highly stratified during decomposition. ISME Journal.

[ref-2] Broeckling CD, Broz AK, Bergelson J, Manter DK, Vivanco JM (2008). Root exudates regulate soil fungal community composition and diversity. Applied and Environmental Microbiology.

[ref-3] Caruso T, Chan Y, Lacap DC, Lau MCY, Mckay CP, Pointing SB (2011). Stochastic and deterministic processes interact in the assembly of desert microbial communities on a global scale. ISME Journal.

[ref-4] Chu H, Sun H, Tripathi BM, Adams JM, Huang R, Zhang Y, Shi Y (2016). Bacterial community dissimilarity between the surface and subsurface soils equals horizontal differences over several kilometers in the western Tibetan Plateau. Environmental Microbiology.

[ref-5] Cline LC, Zak DR (2014). Dispersal limitation structures fungal community assembly in a long-term glacial chronosequence. Environmental Microbiology.

[ref-6] Cole JR, Wang Q, Cardenas E, Fish J, Chai B, Farris RJ, Kulam-Syed-Mohideen A, McGarrell DM, Marsh T, Garrity GM (2009). The ribosomal database project: improved alignments and new tools for rRNA analysis. Nucleic Acids Research.

[ref-7] Davey ML, Heegaard E, Halvorsen R, Ohlson M, Kauserud H (2012). Seasonal trends in the biomass and structure of bryophyte-associated fungal communities explored by 454 pyrosequencing. New Phytologist.

[ref-8] Dumbrell AJ, Nelson M, Helgason T, Dytham C, Fitter AH (2010). Relative roles of niche and neutral processes in structuring a soil microbial community. ISME Journal.

[ref-9] Edgar R (2013). UPARSE: highly accurate OTU sequences from microbial amplicon reads. Nature Methods.

[ref-10] Fierer N, Leff JW, Adams BJ, Nielsen UN, Bates ST, Lauber CL, Owens SM, Gilbert JA, Wall DH, Caporaso JG (2012). Cross-biome metagenomic analyses of soil microbial communities and their functional attributes. Proceedings of the National Academy of Sciences of the United States of America.

[ref-11] Finlay BJ (2002). Global dispersal of free-living microbial eukaryote species. Science.

[ref-12] Ge Y, Zhang J-b, Zhang L-m, Yang M, He J-z (2008). Long-term fertilization regimes affect bacterial community structure and diversity of an agricultural soil in northern China. Journal of Soils and Sediments.

[ref-13] Gilbert JA, Steele JA, Caporaso JG, Steinbruck L, Reeder J, Temperton B, Huse S, McHardy AC, Knight R, Joint I, Somerfield P, Fuhrman JA, Field D (2012). Defining seasonal marine microbial community dynamics. ISME Journal.

[ref-14] Goss-Souza D, Mendes LW, Borges CD, Baretta D, Tsai SM, Rodrigues JLM (2017). Soil microbial community dynamics and assembly under long-term land use change. FEMS Microbiology Ecology.

[ref-15] Hanson CA, Fuhrman JA, Hornerdevine MC, Martiny JBH (2012). Beyond biogeographic patterns: processes shaping the microbial landscape. Nature Reviews Microbiology.

[ref-48] Hu J, Meng D, Liu X, Liang Y, Yin H, Liu H (2018). Response of soil fungal community to long-term chromium contamination. Transactions of Nonferrous Metals Society of China.

[ref-16] Hu W, Zhang Q, Tian T, Li D, Cheng G, Mu J, Wu Q, Niu F, Stegen JC, An L, Feng H (2015). Relative roles of deterministic and stochastic processes in driving the vertical distribution of bacterial communities in a permafrost core from the Qinghai-Tibet Plateau, China. PLOS ONE.

[ref-17] Jiang S, Liu Y, Luo J, Qin M, Johnson NC, Opik M, Vasar M, Chai Y, Zhou X, Mao L (2018). Dynamics of arbuscular mycorrhizal fungal community structure and functioning along a nitrogen enrichment gradient in an alpine meadow ecosystem. New Phytologist.

[ref-18] Kinnunen M, Dechesne A, Albrechtsen HJ, Smets BF (2018). Stochastic processes govern invasion success in microbial communities when the invader is phylogenetically close to resident bacteria. ISME Journal.

[ref-19] Kong Y (2014). Btrim: a fast, lightweight adapter and quality trimming program for next-generation sequencing technologies. Genomics.

[ref-20] Leblanc N, Kinkel LL, Kistler HC (2015). Soil fungal communities respond to grassland plant community richness and soil edaphics. Microbial Ecology.

[ref-21] Li X, Meng D, Li J, Yin H, Liu H, Liu X, Cheng C, Xiao Y, Liu Z, Yan M (2017). Response of soil microbial communities and microbial interactions to long-term heavy metal contamination. Environmental Pollution.

[ref-22] Lipson DA, Schadt CW, Schmidt SK (2002). Changes in soil microbial community structure and function in an alpine dry meadow following spring snow melt. Microbial Ecology.

[ref-23] Magoc T, Salzberg SL (2011). FLASH: fast length adjustment of short reads to improve genome assemblies. Bioinformatics.

[ref-24] Meng D, Li J, Liu T, Liu Y, Yan M, Hu J, Li X, Liu X, Liang Y, Liu H, Yin H (2019). Effects of redox potential on soil cadmium solubility: insight into microbial community. Journal of Environmental Sciences (China).

[ref-25] Pereira e Silva MC, Dias AC, van Elsas JD, Salles JF (2012). Spatial and temporal variation of archaeal, bacterial and fungal communities in agricultural soils. PLOS ONE.

[ref-26] Schadt CW, Martin AP, Lipson DA, Schmidt SK (2003). Seasonal dynamics of previously unknown fungal lineages in tundra soils. Science.

[ref-27] Schoch CL, Shoemaker RA, Seifert KA, Hambleton S, Spatafora JW, Crous PW (2006). A multigene phylogeny of the Dothideomycetes using four nuclear loci. Mycologia.

[ref-28] Segata N, Izard J, Waldron L, Gevers D, Miropolsky L, Garrett WS, Huttenhower C (2011). Metagenomic biomarker discovery and explanation. Genome Biology.

[ref-29] Sheng R, Meng D, Wu M, Di H, Qin H, Wei W (2013). Effect of agricultural land use change on community composition of bacteria and ammonia oxidizers. Journal of Soils and Sediments.

[ref-30] Stegen JC, Freestone AL, Crist TO, Anderson MJ, Chase JM, Comita LS, Cornell HV, Davies KF, Harrison SP, Hurlbert AH, Inouye BD, Kraft NJB, Myers JA, Sanders NJ, Swenson NG, Vellend M, Evans K (2013a). Stochastic and deterministic drivers of spatial and temporal turnover in breeding bird communities. Global Ecology and Biogeography.

[ref-31] Stegen JC, Lin X, Fredrickson JK, Chen X, Kennedy DW, Murray CJ, Rockhold ML, Konopka A (2013b). Quantifying community assembly processes and identifying features that impose them. ISME Journal.

[ref-32] Stegen JC, Lin X, Fredrickson JK, Konopka A (2015). Estimating and mapping ecological processes influencing microbial community assembly. Frontiers in Microbiology.

[ref-33] Stegen JC, Lin X, Konopka AE, Fredrickson JK (2012). Stochastic and deterministic assembly processes in subsurface microbial communities. ISME Journal.

[ref-34] Sun R, Dsouza M, Gilbert JA, Guo X, Wang D, Guo Z, Ni Y, Chu H (2016). Fungal community composition in soils subjected to long‐term chemical fertilization is most influenced by the type of organic matter. Environmental Microbiology.

[ref-35] Tiemann LK, Grandy AS, Atkinson EE, Marin-Spiotta E, McDaniel MD (2015). Crop rotational diversity enhances belowground communities and functions in an agroecosystem. Ecology Letters.

[ref-36] Vellend M (2010). Conceptual synthesis in community ecology. Quarterly Review of Biology.

[ref-37] Voriskova J, Brabcova V, Cajthaml T, Baldrian P (2014). Seasonal dynamics of fungal communities in a temperate oak forest soil. New Phytologist.

[ref-38] Waldrop MP, Zak DR, Blackwood CB, Curtis CD, Tilman D (2006). Resource availability controls fungal diversity across a plant diversity gradient. Ecology Letters.

[ref-39] Wang Q, Garrity GM, Tiedje JM, Cole JR (2007). Naive Bayesian classifier for rapid assignment of rRNA sequences into the new bacterial taxonomy. Applied and Environmental Microbiology.

[ref-40] Wang J, Shen J, Wu Y, Tu C, Soininen J, Stegen JC, He J, Liu X, Zhang L, Zhang E (2013). Phylogenetic beta diversity in bacterial assemblages across ecosystems: deterministic versus stochastic processes. ISME Journal.

[ref-41] Webb CO, Ackerly DD, McPeek MA, Donoghue MJ (2002). Phylogenies and community ecology. Annual Review of Ecology and Systematics.

[ref-42] Xu L, Ravnskov S, Larsen J, Nilsson RH, Nicolaisen M (2012). Soil fungal community structure along a soil health gradient in pea fields examined using deep amplicon sequencing. Soil Biology & Biochemistry.

[ref-43] Yan Q, Stegen JC, Yu Y, Deng Y, Li X, Wu S, Dai L, Zhang X, Li J, Wang C, Ni J, Li X, Hu H, Xiao F, Feng W, Ning D, He Z, Van Nostrand JD, Wu L, Zhou J (2017). Nearly a decade-long repeatable seasonal diversity patterns of bacterioplankton communities in the eutrophic Lake Donghu (Wuhan, China). Molecular Ecology.

[ref-44] Zak DR, Holmes WE, White DC, Peacock AD, Tilman D (2003). Plant diversity, soil microbial communities, and ecosystem function: are there any links?. Ecology.

[ref-45] Zhen Z, Liu H, Wang N, Guo L, Meng J, Ding N, Wu G, Jiang G (2014). Effects of manure compost application on soil microbial community diversity and soil microenvironments in a temperate cropland in China. PLOS ONE.

[ref-46] Zhou J, Kang S, Schadt CW, Garten CT (2008). Spatial scaling of functional gene diversity across various microbial taxa. Proceedings of the National Academy of Sciences of the United States of America.

[ref-47] Zhou J, Ning D (2017). Stochastic community assembly: does it matter in microbial ecology?. Microbiology and Molecular Biology Reviews.

