# Supplementary material for: Stochastic and deterministic drivers of seasonal variation of fungal community in tobacco field soil
Source: PeerJ. 2019 Jun 14;7:e6962. doi: 10.7717/peerj.6962 (PMC6573846; doi:10.7717/peerj.6962)
Supplement: Supplemental Information 2 — WC: water content, OM: organic matter, TN: total nitrogen, Temp: Temperature, S.OTU: observed OTU number. [file peerj-07-6962-s002.docx]

*Supplementary file for*

**Stochastic and deterministic drivers of seasonal variation of fungal community in tobacco field soil**

Xing Li^1,4#^, Tianming Li^1,4#^, Delong Meng^1,4#^, Tianbo Liu^2^, Yongjun Liu^2^, Huaqun Yin^1,4^, Songrong Zeng^3^, Li Shen^1,4^

^1^ School of Minerals Processing and Bioengineering, Central South University, Changsha, Hunan, China.

^2^ College of Agronomy, Hunan Agricultural University, Changsha, Hunan, China.

^3^ Hery Fok Collge of Life Sciences, Shaoguan University, Shaoguan, Guangzhou China

^4^ Key Laboratory of Biometallurgy, Ministry of Education, Changsha, Hunan, China.

**Corresponding Author:**

Songrong Zeng^3^

Hery Fok Collge of Life Sciences, Shaoguan University, Shaguan, 512005, China

Email address: zengsr@sgu.edu.cn．

Li Shen^1,4^

932 Lushan South Street, Yuelu District, Changsha, 410083, China

Email address: lishen@csu.edu.cn.

Table S1 Pearson correlation between soil properties and fungal community diversity indices. WC: water content, OM: organic matter, TN: total nitrogen, Temp: Temperature, S.OTU: observed OTU number.

|  |  | S.OTU | Shannon | invsimpson | Simpson | Pielou | S.chao1 |
| --- | --- | --- | --- | --- | --- | --- | --- |
| WC | Pearson | **-0.868** | **-0.926** | **-0.779** | **-0.912** | **-0.926** | **-0.816** |
|  | *p* | **<0.001** | **<0.001** | **<0.001** | **<0.001** | **<0.001** | **<0.001** |
| OM | Pearson | **0.718** | **0.781** | **0.71** | **0.771** | **0.784** | **0.683** |
|  | *p* | **<0.001** | **<0.001** | **<0.001** | **<0.001** | **<0.001** | **0.001** |
| TN | Pearson | **0.498** | **0.611** | **0.53** | **0.624** | **0.621** | **0.451** |
|  | *p* | **0.025** | **0.004** | **0.016** | **0.003** | **0.003** | **0.046** |
| pH | Pearson | 0.173 | 0.072 | -0.025 | 0.098 | 0.064 | 0.2 |
|  | *p* | 0.465 | 0.762 | 0.915 | 0.680 | 0.788 | 0.399 |
| Temp | Pearson | **-0.844** | **-0.906** | **-0.778** | **-0.882** | **-0.906** | **-0.786** |
|  | *p* | **<0.001** | **<0.001** | **<0.001** | **<0.001** | **<0.001** | **<0.001** |
| Ca | Pearson | 0.042 | 0.097 | 0.188 | 0.082 | 0.099 | 0.004 |
|  | *p* | 0.86 | 0.683 | 0.428 | 0.731 | 0.677 | 0.986 |
| Mn | Pearson | 0.091 | 0.029 | 0.108 | -0.004 | 0.014 | 0.116 |
|  | *p* | 0.703 | 0.903 | 0.651 | 0.987 | 0.954 | 0.625 |
| Fe | Pearson | 0.299 | 0.346 | 0.235 | 0.381 | 0.351 | 0.233 |
|  | *p* | 0.201 | 0.135 | 0.319 | 0.097 | 0.129 | 0.322 |
